# A novel technique for atraumatic transurethral catheterisation of male rats

**DOI:** 10.1242/bio.060476

**Published:** 2024-08-30

**Authors:** Steven Liben Zhang, Allen Wei-Jiat Wong

**Affiliations:** ^1^ Plastic, Reconstructive and Aesthetic Surgery Service, Department of General Surgery, Woodlands Health, Singapore 737628, Singapore; ^2^ Plastic, Reconstructive & Aesthetic Surgery Service, Sengkang General Hospital, Singapore 544886, Singapore

**Keywords:** Atraumatic, Catheterisation, Catheterization, Microscope, Rat, Transurethral

## Abstract

Transurethral catheterisation of male rats is technically difficult owing to anatomical peculiarities. In the male rat, the urethral striated sphincter consists of two lateral fascicles separated by an anterior and a posterior strip of connective tissue, which impedes the smooth insertion of a urinary catheter. For rat studies requiring continuous collection of urine, bladder irrigation, or measurement of bladder pressure, investigators either have to exclude the male population (be limited to the female population) or perform percutaneous (suprapubic) bladder puncture in male rats, which is more traumatic and invasive than transurethral catheterisation. This paper describes a novel, atraumatic method of transurethral catheterisation in the male rat, with the aid of a microscope and microsurgical instruments. Six Wistar rats were used for this experiment, all of which were catheterised successfully, with no evidence of bladder or urethral injury. The study shows that male rats can be safely catheterised via the urethra with the aid of a microscope and microsurgical instruments for both visual and tactile feedback. This is a relatively straightforward technique to learn and can allow for inclusion of male rats in future studies requiring urinary analysis or bladder irrigation, without the need for traumatic percutaneous (suprapubic) bladder puncture.

## INTRODUCTION

Transurethral catheterisation is a critical procedure for any study requiring collection of urine, bladder irrigation, or measurement of bladder pressure. Most of such studies involving rodents are limited exclusively to the female sex due to differences between the male and female lower urinary tract anatomy (Lamanna et al., 2020). In particular, the male urethra has a striated sphincter consisting of two lateral fascicles separated by an anterior and a posterior strip of connective tissue, which make male rat catheterisation particularly challenging (Biérinx and Sebille, 2006; Thai et al., 2010; Lee et al., 2017). The smaller diameter of the male urethra also makes it susceptible to iatrogenic injury or rupture during attempts at catheterising. In current literature, if a research protocol requires the continuous collection of urine specimen, bladder irrigation, or measurement of bladder pressure in rats, the male sex is usually excluded, because the alternative involves percutaneous (suprapubic) bladder puncture which is more traumatic and invasive than transurethral catheterisation, with the potential for visceral injury, bladder rupture or even death. In order to overcome this difficulty in collecting urine specimens from male rats, we describe a novel and atraumatic method of transurethral catheterisation in the male rat, using an operative microscope and microsurgical instruments.

## RESULTS AND DISCUSSION

All six rats were catheterised successfully, with presence of Methylene Blue within the bladder and urethra only, proving that there was no evidence of injury to the bladder wall or urethra. In this study, we report a novel and atraumatic method of transurethral catheterisation in the male rat. The use of a microscope and microsurgical instruments allows for instrumentation of the male urethra, which is hitherto avoided in view of its smaller size and anatomical complexity. This would potentially facilitate the inclusion of the male rat population in urinary studies, while avoiding the traumatic and invasive alternative of percutaneous (suprapubic) bladder puncture.

Our technique addressed the two main difficulties in catheterising the male rat. Firstly, the small calibre of the male urethra is addressed by gradual dilatation of the urethra opening with soft cannula sheaths with the aid of the microscope. This allowed the eventual insertion of the 2.7Fr paediatric vascular catheter into the external urethra opening. Secondly, the ventral plates in the urethra were overcome by manipulation of the penis to change the angle of entry by the catheter. To avoid injury from excessive force, the use of microsurgical jeweller's forceps also prevented the operator from exerting too much force during catheter advancement. The operator would also be able to feel the tactile feedback through the fine tips of the jeweller's forceps during the advancement of the catheter.

The potential applications of this technique are numerous, including any study that requires collection of urine, bladder irrigation, or measurement of bladder pressure. These could range from bladder inoculation of bacteria for the study of urinary tract infections (Hung et al., 2009); measurement of bladder pressures for the study of urethral function and its relation to stress urinary incontinence (Janssen et al., 2021); and assessment of bladder tissue by flow cytometry for the study of host responses to infection and/or cancer (Scharff et al., 2017; Hadaschik et al., 2007), among others.

To our knowledge, there have only been two other descriptions of transurethral instrumentation in the male rat to date (Lee et al., 2017; Lamanna et al., 2020). The first description (Lee et al., 2017) is potentially traumatic as it involves the use of a needle that enters the distal urethra, and is only described for single use instillation of fluids (as opposed to the placement of an in-dwelling catheter, which can allow for repeated collection of urine samples and/or continuous urine output monitoring). The second description (Lamanna et al., 2020) uses a technique similar to the former, and also relies on a customised catheter design involving multiple separate components that are cut and combined manually; this may not be easily reproducible and/or accessible.

There are some limitations in our study. First, a surgical microscope, microsurgical instruments, and paediatric vascular catheter may not be readily available to the investigator. This may be easily overcome by collaborating with affiliated hospitals or medical institutions; or practicing the same technique with laboratory microscopes and similar fine instruments. We believe that these limitations can be easily overcome and will be easily applied in future studies.

### Conclusions

Male rats can be safely catheterised via the urethra with the aid of a microscope and microsurgical instruments for both visual and tactile feedback. This is a relatively straightforward technique to learn and can allow for inclusion of male rats in future studies requiring urinary analysis or bladder irrigation, without the need for traumatic percutaneous (suprapubic) bladder puncture.

## MATERIALS AND METHODS

All experimental protocols were approved by the Singhealth Institutional Animal Care and Use Committee (IACUC) and performed in accordance with their guidelines. Six Wistar rats were used for this study. All rats were anesthetized with 2.5% isoflurane via inhalation through a nose cone. Surgical microscopes (Leica M50 Stereo Microscope, Wetzlar, Germany) were used at ×6 magnification for clear visualisation of the external urethral orifice. Transurethral catheterisation was performed using the following steps:

(1) The prepuce was retracted with a pair of microsurgical jeweller's forceps to expose the external urethral orifice
(2) A ventral urethral plate with a mid-line ridge over the distal urethra was identified and avoided
(3) The distal urethral tract was sequentially dilated via insertion of a 24G intravenous catheter sheath (Introcan Safety^®^, B. Braun), which was subsequently removed and followed by insertion of a 22G intravenous catheter sheath (Introcan Safety^®^, B. Braun), which was subsequently removed (Fig. S1).
(4) Transurethral catheterisation was performed with a modified 2.7 Fr paediatric vascular catheter (Cook Medical, Indiana, USA). The catheter was held by a pair of jeweller's forceps, and advanced carefully. If any resistance was felt, the catheter would be withdrawn and repositioned again. The penis was manipulated sequentially to facilitate atraumatic insertion following the natural direction of the urethra at different portions, as follows (Fig. S2):

(a) For the first centimetre, the catheter was inserted with the penis in an upright position.
(b) For the second centimetre, the catheter was advanced with the penis lying flat on the abdomen.
(c) For the third centimetre, the catheter was advanced with the penis held on a stretch directed caudally and parallel to the horizontal plane.

These manoeuvres were necessary to circumvent the convoluted path that the urethra takes around the pelvis.

**Fig. 1.**
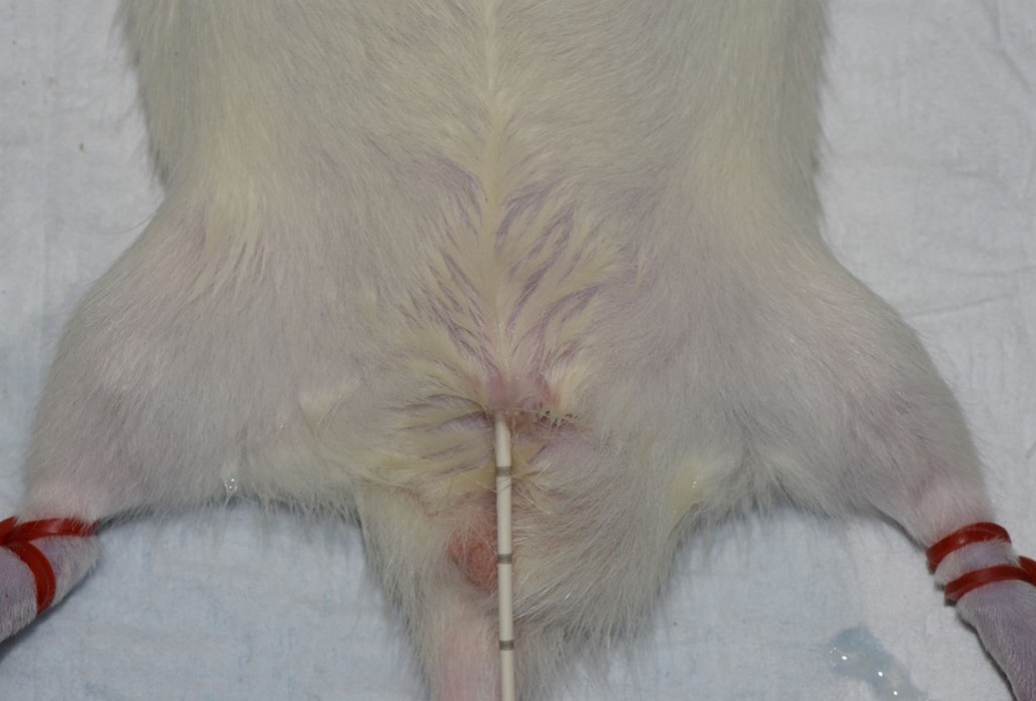
Successful placement of urinary catheter in male rat.

**Fig. 2.**
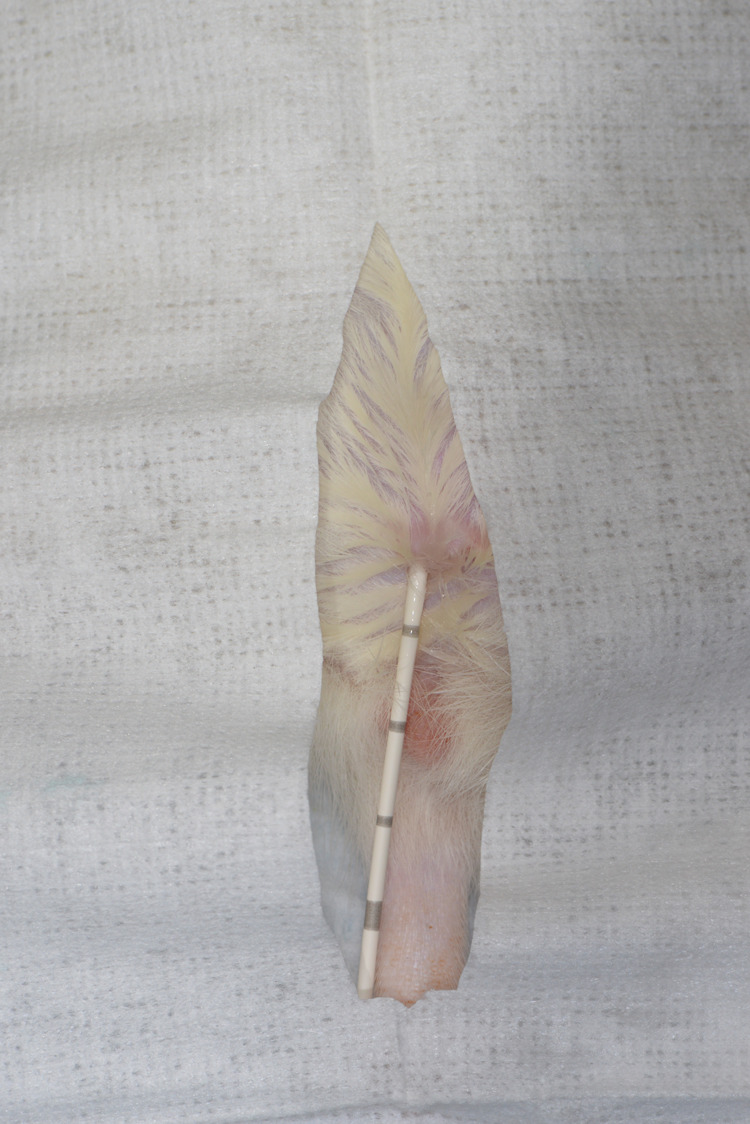
Close-up photo of urinary catheter placement.

Figs 1 and 2 show the placement of the urinary catheter following successful catheterisation.

**Fig. 3.**
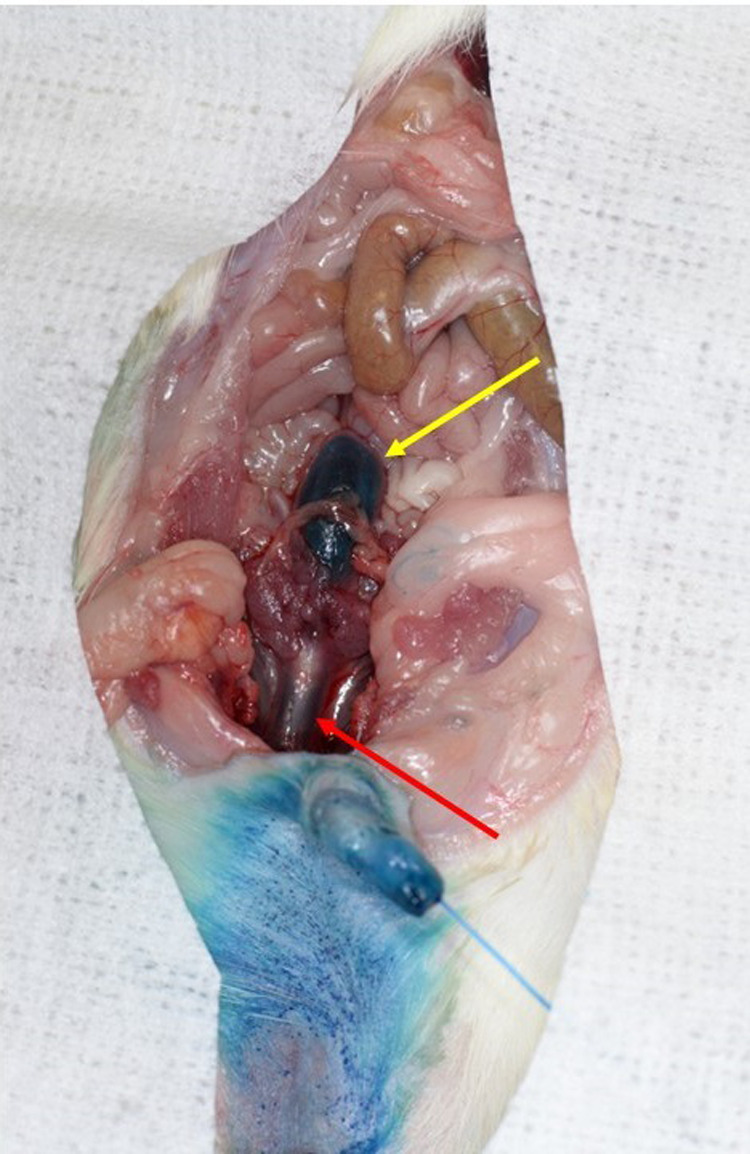
**Laparotomy showing presence of methylene blue within the urinary bladder (yellow arrow) and urethra (red arrow) only.** This proves that the urinary catheter was successfully inserted into the urinary bladder without causing injury to the bladder wall or urethra.

Methylene Blue was then injected through the urinary catheter into the urinary bladder. A laparotomy was performed to check for presence of Methylene Blue within the bladder and urethra, and any urethral injury that would be evidenced by Methylene Blue leaks. Fig. 3 shows the presence of Methylene Blue in the urinary bladder and urethra only, which proves that the urinary catheter was successfully inserted into the urinary bladder without causing injury to the bladder wall or urethra.

**Fig. 4.**
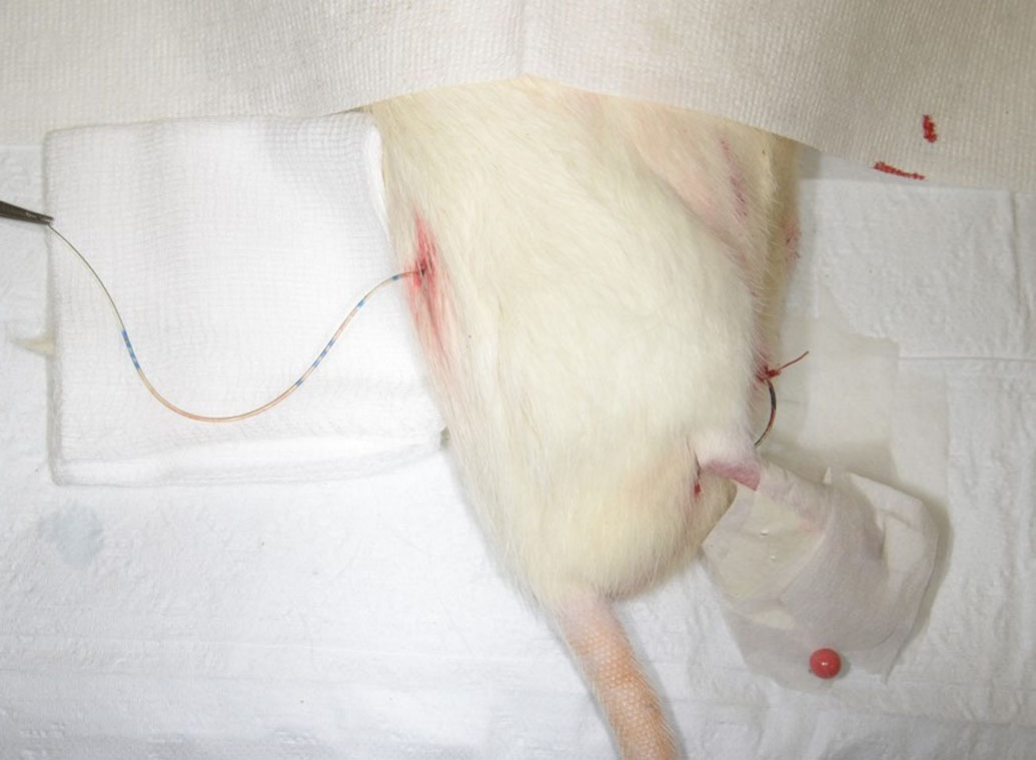
Placement of urinary catheter on dorsum of rat (through a subcutaneous tunnel), to allow for easier nursing.

Fig. 4 shows the passage of the urinary catheter through a subcutaneous tunnel for placement at the dorsum of the rat, to prevent the rat from lying on or gnawing on it, and hence allow for easier nursing.

## Supplementary Material

10.1242/biolopen.060476_sup1 Supplementary information
