# Supplementary material for: A novel technique for atraumatic transurethral catheterisation of male rats
Source: Biol Open. 2024 Aug 30;13(9):bio060476. doi: 10.1242/bio.060476 (PMC11381925; doi:10.1242/bio.060476)
Supplement: Supplementary information [file biolopen-13-060476-s1.pdf]

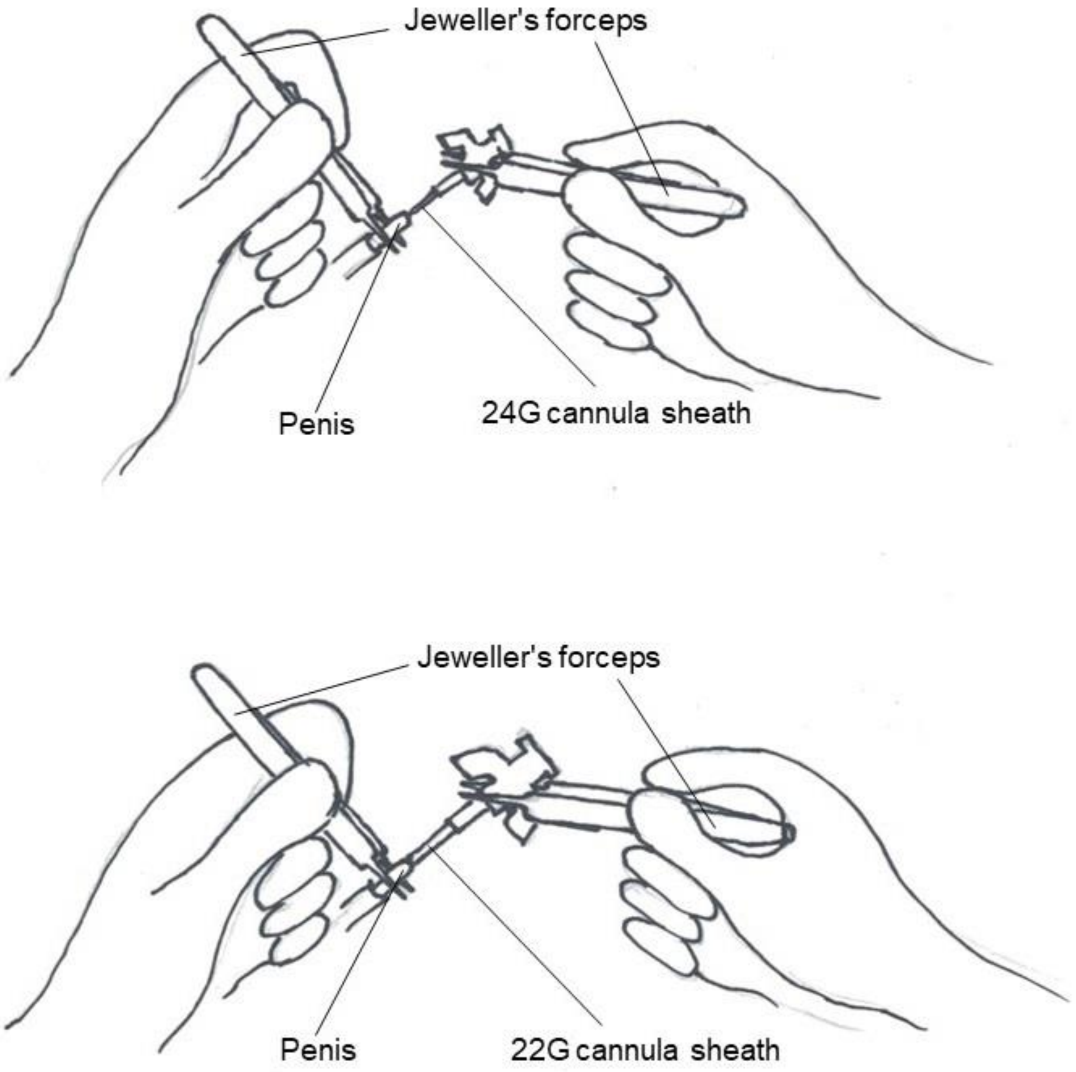

Fig. S1. Sequential dilation of the distal urethral tract with a 24G intravenous cannula sheath (top), followed by a 22G intravenous cannula sheath (bottom).

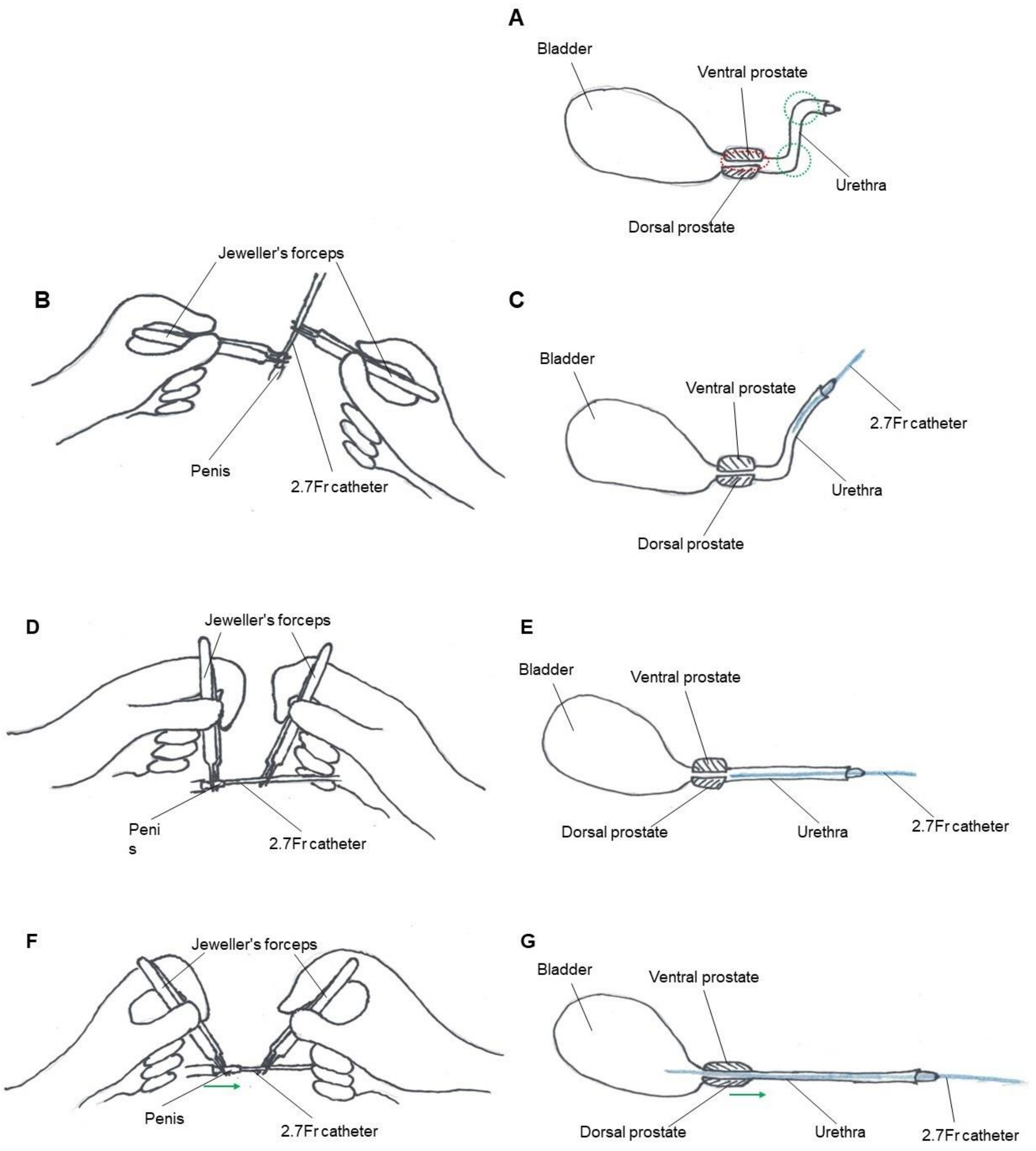

Fig. S2. Illustrations of catheterisation procedure (left) and corresponding urethral anatomy (right). **A**: simplified natural course of the urethra at rest, with two significant 'bends' (green circles), and narrowing at the prostate (red circle). **B, C**: initial insertion of the catheter with penis in upright position for the first centimetre, which straightens the first 'bend' of the urethra. **D, E**: subsequent insertion of the catheter with the penis lying flat / parallel to the abdomen for the second centimetre, which straightens the next 'bend' of the urethra. **F, G**: the last part / centimetre of insertion is performed with the penis held on stretch directed caudally (green arrow), which makes the narrowing of the prostatic urethra more gradual.

Biology Open • Supplementary information
